# 

**Published:** 1885-02

**Authors:** 


### INTERNATIONAL MEDICAL CONGRESS.

The ninth triennial session of this body will meet in Washington, in 1887, and the committee is busily at work perfecting the general plan and the permanent organization. The meeting of 1881, in London, was attended by a large number of Americans, and the feeling among the delegates was, that when it should convene in this country, every effort must be made to secure as good or better results than were there attained. The committee has presented a partial report to the medical profession, the sections have been designated, and the general officers selected. That it may be known who are eligible to membership we publish the first two rules, which read as follows:

1. The congress will be composed of members of the regular medical profession, who shall have inscribed their names on the register of the congress, and shall have taken out their tickets of admission. As regards foreign members, the above conditions are the only ones which it seems expedient to impose.

The American members of the congress shall be appointed by the American Medical Association, by regularly organized State and local medical societies, and also by such general organizations relating to special departments and purposes as the American Academy of Medicine, the American Surgical Association, the American Gynecological, Ophthalmological, Otological, Laryngological, Neurological, and Dermatological Societies, and the American Public Health Association; each of the foregoing societies being entitled to appoint one delegate for every ten of their [its] membership.

2. The work of the congress is divided into eighteen sections, as follows, viz: 1. Medical Education, Legislation and Registration, including methods of teaching and buildings, apparatus, etc., connected therewith. 2. Anatomy. 3. Physiology. 4. Pathology. 5. Medicine. 6. Surgery. 7. Obstetrics. 8. Gynecology. 9. Ophthalmology. 10. Otology. 11. Dermatology and Syphilis. 12. Nervous Diseases and Psychiatry. 13. Laryngology. 14. Public and International Hygiene. 15. Collective Investigation, Nomenclature and Vital Statistics. 16. Military and Naval Surgery and Medicine. 17. Experimental Therapeutics and Pharmacology. 18. Diseases of Children.

It may be well to compare with these the division into sections of the London congress, which was as follows:

1. Anatomy. 2. Physiology. 3. Pathology. 4. Medicine. 4. (sub-section) Diseases of the Throat. 5. Surgery. 6. Obstetric Medicine and Surgery. 7. Diseases of Children. 8. Mental Diseases. 9. Ophthalmology. 10. Diseases of the Ear. 11. Diseases of the Skin. 12. Diseases of the Teeth. 13. State Medicine. 14. Military Surgery and Medicine. 15. Materia Medica and Pharmacology.

It will be seen that so far in the plan proposed for the American meeting, no provision has been made for a section of diseases of the teeth. We think a great mistake was made in not recognizing this specialty at the beginning. At the London meeting there were in attendance two hundred and twenty delegates from America, which was nineteen more than were registered from France, seventeen more than from Germany, and one hundred and eighty-seven more than from Russia, which had the next largest representation. Of the American delegates, practitioners of dentistry formed an important percentage, and the sessions of section twelve were not exceeded in interest by those of any of the divisions of the congress. In no country upon the face of the globe does dentistry occupy a higher position than in America; and were our fifteen thousand dentists to be denied recognition, it would not only be a professional, but a national humiliation. Those dentists who were in attendance upon the London meeting have a great debt of hospitality which they desire an opportunity to repay, and it will be the source of much bitter feeling if the privilege be denied them. Nor can we see how with any kind of consistency they can be excluded. The meeting will be under the auspices of the American Medical Association, and this body has a section of dental and oral surgery, and in view of the precedent established in 1881, such a division of the congress of 1887 should have been determined upon at the outset.

The attention of the committee has been called to the omission, and there is little doubt that it will be remedied, and a section of oral medicine and surgery organized. The whole power and authority rests with the general committee to name the officers and put such a section in working order, and this will, in the event of its establishment, forestall any charges of seeking position for personal aggrandizement.

As a medical body it is, of course, quite impossible for the congress to recognize any but the one distinguishing medical degree of M. D. A reference to the first rule quoted will convince any one of this. "The congress will be composed of members of *the regular medical profession.*" This is more restrictive than the qualification prescribed by the London meeting, whose rule read: "The congress will be composed of *medical men legally qualified to practice* in their respective countries." No degree other than a regular medical one was there recognized, yet practically none were shut out, for upon registering, when asked "what is your title," it was necessary but to answer "simply doctor." In some cases, when other than medical degrees were offered, the application was refused, but it was only requisite that the applicant should appear at the desk a little later, and to the stereotyped query give the title of "doctor." It is probable that some such opportunity for membership will be given in 1887. Certainly a section of diseases of the teeth would be comparatively inefficient if none but those possessed of a strictly medical degree were admitted.

The officers of the congress selected are as follows:

President—Dr. Austin Flint, Sen., of New York.

Vice-Presidents—Dr. Alfred Stillé, of Philadelphia; Dr. Henry I. Bowditch, of Boston; Dr. R. P. Howard, of Montreal, Canada.

Secretary-General—Dr. J. S. Billings, U. S. A.

Treasurer—Dr. J. M. Browne, U. S. Navy.

Members of the Executive Committee (in addition to the President, Secretary-General and Treasurer)—Dr. I. Minis Hays, Philadelphia; Dr. A. Jacobi, New York; Dr. Christopher Johnston, Baltimore; Dr. S. C. Busey, Washington; and the chairman of the different sections.

### The Independent Practitioner—Vol. VI.

### PUBLISHED BY DENTISTS FOR DENTISTS.

### Prospectus.

This Journal is published by an association of professional men who have no ulterior objects other than to afford to the profession an independent, outspoken Journal, which shall be the exponent of higher aims and a better practice.

Having no connection with any school, clique, or advertising firm, it will be impartial in its judgment of professional matters, and its appeals for support are directed to those who believe that a liberal profession should have an independent literature.

While not neglecting practical details, it will pay especial attention to those oral diseases that have usually been under the care of the general practitioner, but which more properly belong to the specialty of dentistry.

Its pages will be open to any member of the profession who has anything of interest to communicate, and it invites a free expression of views on all subjects medical or dental.

As each of the gentlemen forming the association is practically a member of the editorial corps, it will have abundant opportunity for collecting all the latest professional news and intelligence.

It will have a body of regular contributors, selected from the very best writers in the profession, and will thus be certain of an abundance of interesting original matter.

It will pay especial attention to reports of the meetings of dental societies, and its aim will be to publish an epitome of all important discussions of professional matters.

It will contain original abstracts and translations of all that is of moment in the foreign journals.

Its profits, if any, will be devoted to its improvement by adding needed illustrations, paying for valuable original communications, and enlarging its size whenever this becomes advisable.

To the accomplishment of the work thus outlined (necessarily relying upon the hearty approval of an appreciative profession), each of the publishers of the Independent Practitioner has pledged his best efforts.

Editorial communications should be addressed to Dr. W. C. Barrett, No. 11 West Chippewa St., Buffalo, N. Y. Send all business letters to Dr. William Carr, 35 West Forty-Sixth Street, New York.

| | | |
|---|---|---|
| ABBOTT, FRANK....New York. | CARR, WILLIAM..New York. | JARVIE, WM. Jr..Brooklyn. |
| BARRETT, W. C.........Buffalo. | FRANCIS, C. E....New York. | NORTHROP, A. L.New York. |
| BODECKER, C. F. W..New York. | HILL, O. E..........Brooklyn. | PALMER, S. B......Syracuse. |

W. D. MILLER, Berlin, Germany.

